# Antimicrobial Nonsusceptibility of Gram-Negative Bloodstream Isolates, Veterans Health Administration System, United States, 2003–2013^1^

**DOI:** 10.3201/eid2311.161214

**Published:** 2017-11

**Authors:** Michihiko Goto, Jennifer S. McDanel, Makoto M. Jones, Daniel J. Livorsi, Michael E. Ohl, Brice F. Beck, Kelly K. Richardson, Bruce Alexander, Eli N. Perencevich

**Affiliations:** Iowa City Veterans Affairs Health Care System, Iowa City, Iowa, USA (M. Goto, J.S. McDanel, D.J. Livorsi, M.E. Ohl, B.F. Beck, K.K. Richardson, B. Alexander, E.N. Perencevich);; University of Iowa Carver College of Medicine, Iowa City (M. Goto, J.S. McDanel, D.J. Livorsi, M.E. Ohl, E.N. Perencevich);; Salt Lake City Veterans Affairs Health Care System, Salt Lake City, Utah, USA (M.M. Jones);; University of Utah School of Medicine, Salt Lake City USA (M.M. Jones)

## Abstract

Bacteremia caused by gram-negative bacteria is associated with serious illness and death, and emergence of antimicrobial drug resistance in these bacteria is a major concern. Using national microbiology and patient data for 2003–2013 from the US Veterans Health Administration, we characterized nonsusceptibility trends of community-acquired, community-onset; healthcare-associated, community-onset; and hospital-onset bacteremia for selected gram-negative bacteria (*Escherichia coli*, *Klebsiella* spp., *Pseudomonas aeruginosa*, and *Acinetobacter* spp.). For 47,746 episodes of bacteremia, the incidence rate was 6.37 episodes/10,000 person-years for community-onset bacteremia and 4.53 episodes/10,000 patient-days for hospital-onset bacteremia. For *Klebsiella* spp., *P. aeruginosa*, and *Acinetobacter* spp., we observed a decreasing proportion of nonsusceptibility across nearly all antimicrobial drug classes for patients with healthcare exposure; trends for community-acquired, community-onset isolates were stable or increasing. The role of infection control and antimicrobial stewardship efforts in inpatient settings in the decrease in drug resistance rates for hospital-onset isolates needs to be determined.

Despite advances in public health and medical care, bacteremia is still a major cause of illness and death (*1*–*3*). Bacteremia caused by gram-negative bacteria is a frequent cause of severe sepsis and septic shock (*4*,*5*) and poses serious therapeutic challenges. Treatment options are limited because of increased infections with multidrug-resistant, gram-negative bacteria in community and hospital settings. Understanding the epidemiology of infections with gram-negative bacteria is needed to improve guidelines providing empiric therapy recommendations and adequately allocate resources toward infection control and antimicrobial stewardship programs.

Descriptions of the epidemiology of gram-negative bacteremia have been limited by care settings, referral bias, small geographic regions, short study duration, or case identification by use of administrative code data (*6*–*10*). As modern healthcare systems become increasingly diverse and complex, large-scale studies are needed that accurately estimate the burden of antimicrobial resistance and chronologic trends of gram-negative bacteremia (*11*–*13*).

The Veterans Health Administration (VHA) is the largest healthcare system in the United States and has ≈8 million veterans enrolled (*14*). The VHA uses an integrated electronic health record and a nationwide data repository. Microbiological results from all VHA facilities have been added to the data repository, which enables identification of all cases of gram-negative bacteremia across the entire VHA system. These results enable examination of national trends in gram-negative bacteremia in a geographically dispersed and diverse population.

We previously reported incidence rates of community-onset and hospital-onset bacteremia caused by 3 species of gram-negative bacteria in a cohort of patients admitted to the VHA system during 2003–2013 (*15*). In this article, we expand on that study by reporting trends in antimicrobial nonsusceptibility (intermediate or resistant) among bacteremia isolates of 4 selected gram-negative bacteria (*Escherichia coli*, *Klebsiella* spp., *Pseudomonas aeruginosa*, and *Acinetobacter* spp.) in the national VHA healthcare system.

## Methods

### Study Population

We analyzed a retrospective cohort of all veterans who were admitted to acute-care units at VHA hospitals during January 2003–December 2013 and who had positive blood cultures for *E. coli*, *Klebsiella* spp., *P. aeruginosa*, or *Acinetobacter* spp. between 48 hours before admission and time of discharge. We used these 4 bacterial species a priori because studies consistently showed that these organisms represent most gram-negative bacteria causing bacteremia across various healthcare settings (*3*,*16*–*22*). If 1 patient had multiple positive blood cultures for the same species during 1 hospital admission, we included only the first isolate. To estimate the population at risk for community-onset bacteremia, we identified the number of veterans who had visited VHA clinics and emergency departments inside the catchment areas of acute inpatient care facilities by calendar year (outpatient denominator for community-onset bacteremia). To estimate the population at risk for hospital-onset bacteremia, we calculated patient-days in acute inpatient care units (inpatient denominator for hospital-onset bacteremia).

Data for 130 VHA acute-care hospitals in the 48 contiguous states, the District of Columbia, and Puerto Rico were used in this study (*23*). Using the US Department of Agriculture and Department of Health and Human Services Rural-Urban Commuting Areas System, we found that 107 facilities were located in urban areas, 22 in rural areas, and 1 in a highly rural area (*24*). These VA hospitals also serve as referral centers for >1,400 outreach clinics. Acute inpatient capacities were 10–260 beds. Total acute inpatient capacity was ≈10,000 acute-care beds, including 1,900 authorized intensive care unit beds (*25*). We did not include stays at mental health, rehabilitation, and nursing home care units. All but 3 included hospitals have on-site microbiology laboratories, and they are required to conduct quality control/quality assessment routinely per requirements of VHA-designated accreditation organizations and to use methods and equipment approved by the Food and Drug Administration (FDA; Silver Spring, MD, USA).

The institutional review board at the University of the Iowa and the research and development committee of Iowa City Veterans Affairs Health Care System approved this study. A waiver of informed consent was issued for this retrospective analysis.

### Data Source

We obtained data through Veterans Affairs Informatics and Computing Infrastructure, which includes data extracted from the VHA integrated electronic medical record system. Susceptibility results in microbiology reports (susceptible/intermediate/resistant) were recorded in a standardized manner, and isolates were classified as nonsusceptible if they were reported as intermediate resistance or resistant. MICs or sizes of inhibition zones were not typically available.

### Definitions

Bacteremia episodes were classified according to Centers for Diseases Control and Prevention (Atlanta, GA, USA) criteria as community-onset and hospital-onset. This classification was based on time of the first positive blood culture as a standard definition (*26*). 

An episode of bacteremia was considered to be community-onset when the first positive blood culture was collected between 48 hours before and <48 hours after admission. For episodes of hospital-onset bacteremia, the first positive blood culture was collected >48 hours after admission.

We further categorized community-onset bacteremia episodes as community-acquired, community-onset and healthcare-associated, community-onset on the basis of healthcare exposure in the VHA before hospital admission. Episodes were classified as healthcare-associated, community-onset when the patient had been admitted to an acute-care facility <90 days before onset of bacteremia; was a resident of a nursing home or rehabilitation facility; was receiving renal replacement therapy; or received wound care or specialized nursing care in an outpatient setting or at home in the 30 days before onset of bacteremia (*19*). If the patient with community-onset bacteremia did not meet these criteria, the episode was classified as community-acquired, community-onset. Information for healthcare exposure outside the VHA before admission was available only if the VHA was the payer of care.

We categorized antimicrobial drugs listed on susceptibility reports into antimicrobial classes by using interim standard definitions for acquired resistance produced by an international panel of experts from the Centers for Disease Control and Prevention and the European Centre for Disease Prevention and Control (Solna, Sweden) (*27*). We provide included antimicrobial agents and antimicrobial classes for each organism (Technical Appendix Tables 1–3). Isolates were considered nonsusceptible if they were not susceptible to >1 agent in an antimicrobial class (*27*).

### Measurements

We focused primarily on annual proportions and trends of antimicrobial nonsusceptibility rates for the 4 most clinically relevant antimicrobial classes (carbapenems, extended-spectrum cephalosporins, aminoglycosides, and fluoroquinolones) used in gram-negative bacteremia management. We also evaluated incidence rates per 10,000 outpatients for community-onset bacteremia and incidence rates per 10,000 patient-days and per 1,000 admissions for hospital-onset bacteremia for each organism. To enable comparisons with previously reported studies, we calculated age-standardized incidence rates by using a direct method, used US population data for 2000 as a standard population for community-onset bacteremia (*28*), and reported incidence rates with 2 denominators for hospital-onset bacteremia.

### Statistical Analysis

We summarized characteristics of the study population and incidence rates for all 3 categories by using descriptive statistics. We calculated 95% CIs by using the Clopper-Pearson exact method (*29*) for crude incidence rates. Proportions among acquisition categories were compared by using Fisher exact tests. To assess annual trends in proportions of nonsusceptible isolates, we used the Cochrane-Armitage χ^2^ test for trend. All p values were 2-sided, and values <0.05 were considered statistically significant. All statistical analyses were performed by using SAS version 9.4 (SAS Institute Inc., Cary, NC, USA).

## Results

### Study Population Demographics and Overall Incidence Rates

During 2003–2013, the VHA provided 53,576,096 person-years of outpatient care and 30,015,733 patient-days of acute inpatient care. We obtained demographic characteristics of the study population (Table 1). A total of 47,746 episodes of gram-negative bacteremia occurred during the study. Most (96.4%) patients were male, and community-acquired, community-onset cases accounted for 42.3% of cases of gram-negative bacteremia, followed by healthcare-associated, community-onset (29.2%) and hospital-onset (28.5%) (Table 2; Technical Appendix Figure 1).

**Table 1:** Characteristics of acute-care patients, by year, Veterans Health Administration System, United States, 2003–2013

Characteristic	2003	2004	2005	2006	2007	2008	2009	2010	2011	2012	2013
Outpatient care											
No. outpatients	4,357	4,532	4,623	4,705	4,719	4,784	4,945	5,083	5,193	5,281	5,355
Mean age, y, on Jan 1	61.5	61.7	61.7	61.8	61.6	61.4	61.1	61.0	60.9	60.7	60.6
Inpatient care											
No. hospital admissions	470	481	489	492	498	516	536	549	552	552	545
Average length of stay, d	6.0	5.8	5.6	5.5	5.4	5.3	5.1	5.0	5.0	4.8	4.8
No. patient-days	2,805	2,783	2,749	2,718	2,701	2,745	2,747	2,748	2,735	2,675	2,611

**Table 2:** Characteristics of patients with bacteremia caused by gram-negative bacteria, Veterans Health Administration System, United States, 2003–2013*

Characteristic	Total	*Escherichia coli*	*Klebsiella* spp.	*Pseudomonas aeruginosa*	*Acinetobacter* spp.
No.	47,746	24,557	14,270	6,929	1,990
Age, y, mean ± SD	69.1 ± 2.2	69.8 ± 12.3	68.5 ± 12.1	68.5 ± 11.9	65.7 ± 12.6
Sex, %					
M	96.40	95.30	97.30	97.90	97.30
F	3.60	4.70	2.70	2.10	2.70
Infection					
CA-CO	20,210 (42.3)	13,515 (55.0)	4,802 (33.7)	1,454 (21.0)	439 (22.1)
HCA-CO	13,932 (29.2)	6,857 (27.9)	4,242 (29.7)	2,262 (32.7)	571 (28.7)
HO	13,604 (28.5)	4,185 (17.0)	5,226 (36.6)	3,213 (46.4)	980 (49.3)
HO (ICU onset)	3,819 (8.0)	838 (3.4)	1,389 (9.7)	1,174 (16.9)	418 (21.0)

*Values are no. (%) unless otherwise indicated. CA-CO, community-acquired, community-onset; HCA-CO, healthcare-associated, community-onset; HO, hospital-onset; ICU, intensive care unit.

Overall incidence rates from the entire study were 3.77 episodes/10,000 person-years for community-acquired, community-onset gram-negative bacteremia, 2.60 episodes/10,000 person-years for healthcare-associated, community-onset gram-negative bacteremia, and 4.53 episodes/10,000 patient-days (2.40 episodes/1,000 admissions) for hospital-onset gram-negative bacteremia. Age-standardized incidence rates were 1.92 episodes/10,000 person-years for community-acquired, community-onset gram-negative bacteremia and 1.40 episodes/10,000 person-years for healthcare-associated, community-onset gram-negative bacteremia (Technical Appendix Figures 2–4).

### Trends for Antimicrobial Drug Susceptibilities

Susceptibility data were available for >96% of all organism–antimicrobial drug combinations. The only exception was carbapenem susceptibility of *Acinetobacter* spp., which was available for 90% of isolates tested.

#### E. coli

**Figure 1:**
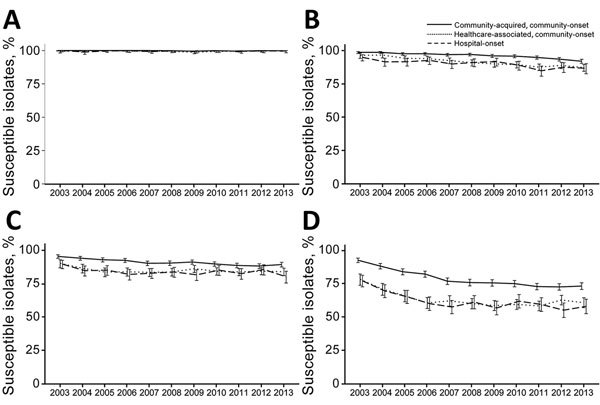
Trends of selected antimicrobial susceptibilities for *Escherichia coli* isolates from patients with bacteremia, Veterans Health Administration System, United States, 2003–2013. A) Carbapenems, B) Extended-spectrum cephalosporins, C) Aminoglycosides, D) Fluoroquinolones. Error bars indicate 95% CIs.

Carbapenem nonsusceptibility for *E. coli* was infrequent (0.3%) (Figure 1). However, nonsusceptibility to carbapenems increased for community-acquired, community-onset isolates (0 in 2003–2007 and 0.2% in 2008–2013, p<0.01 by test for trend). Nonsusceptibility for healthcare-associated, community-onset (p = 0.92 by test for trend) and hospital-onset (p = 0.82 by test for trend) isolates did not change.

Overall, 6.3% of *E. coli* isolates were nonsusceptible to extended-spectrum cephalosporins; an increasing proportion were nonsusceptible (4.1% in 2003–2007 and 7.9% in 2008–2013, p<0.01 by test for trend). Extended-spectrum cephalosporin-nonsusceptible isolates were more frequent for patients with previous or recent healthcare exposure (community-acquired, community-onset 3.9%; healthcare-associated, community-onset 9.0%, and hospital-onset 9.8%, p<0.01). The proportion of isolates that were nonsusceptible to extended-spectrum cephalosporin increased for all categories (community-acquired, community-onset; healthcare-associated, community-onset; hospital-onset, p<0.01 by test for trend).

The overall proportion of isolates that were nonsusceptible to aminoglycosides was 11.9%. Healthcare exposure was associated with higher rates of nonsusceptibility (community-acquired, community-onset 9.0%; healthcare-associated, community-onset 15.6%; hospital-onset 16.0%, p<0.01). We observed increasing nonsusceptibility rates during the study (10.5% in 2003–2007 and 12.9% in 2008–2013, p<0.01 by test for trend).

Incidence rates for fluoroquinolone-nonsusceptible isolates rapidly increased during the first half of the study and then remained stable (23.7% in 2003–2007 and 32.4% in 2008–2013, p<0.01). Fluoroquinolone-nonsusceptible isolates were more frequent in patients with healthcare exposure (community-acquired, community-onset 21.6%; healthcare-associated, community-onset 37.6%; hospital-onset 37.9%, p<0.01), and nonsusceptibility rates increased in groups with these exposures (community-acquired, community-onset; healthcare-associated, community-onset; and hospital-onset, p<0.01 by test for trend).

#### *Klebsiella* spp.

**Figure 2:**
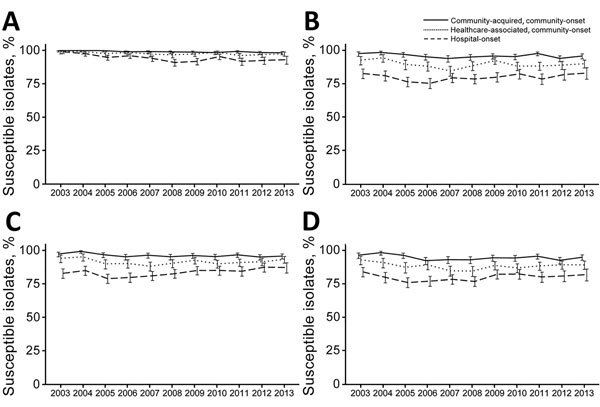
Trends of selected antimicrobial susceptibilities for *Klebsiella* spp. isolates from patients with bacteremia, Veterans Health Administration System, United States, 2003–2013. A) Carbapenems, B) Extended-spectrum cephalosporins, C) Aminoglycosides, D) Fluoroquinolones. Error bars indicate 95% CIs.

Carbapenem nonsusceptibility was reported for 3.0% of *Klebsiella* spp. isolates (Figure 2). We observed a trend toward increasing nonsusceptibility rates (2.1% in 2003–2007 and 3.7% in 2008–2013, p<0.01 by test for trend). Healthcare exposure was associated with higher rates of carbapenem nonsusceptibility (community-acquired, community-onset 0.9%; healthcare-associated, community-onset 2.2%; hospital-onset 5.6%, p<0.01).

Overall, 11.8% of isolates had nonsusceptible results to extended-spectrum cephalosporins and a relatively stable proportion of nonsusceptibility rates (12.6% in 2003–2007 and 11.2% in 2008–2013, p = 0.07 by test for trend). However, nonsusceptibility rates increased for community-acquired, community-onset isolates (p = 0.04 by test for trend) but not for healthcare-associated, community-onset (p = 0.17) and hospital-onset isolates (p = 0.21). Proportions that were nonsusceptible to extended-spectrum cephalosporins were more frequent for healthcare-associated, community-onset (10.4%) and hospital-onset (20.2%) isolates than for community-acquired, community-onset isolates (3.9%, p<0.01).

Overall, 9.8% of isolates were nonsusceptible to aminoglycosides, and a higher proportion were nonsusceptible for patients with healthcare exposure (community-acquired, community-onset 3.6%; healthcare-associated, community-onset 8.5%; hospital-onset 16.7%, p<0.01). Overall, nonsusceptibility to aminoglycosides decreased (10.9% in 2003–2007 and 9.0% in 2008–2013, p<0.01 by test for trend). However, nonsusceptibility increased for community-acquired, community-onset isolates (2.9% in 2003–2007 and 4.1% in 2008–2013, p = 0.01 by test for trend) and decreased for hospital-onset isolates (18.6% in 2003–2007 and 14.8% in 2008–2013, p<0.01 by test for trend).

Fluoroquinolone nonsusceptibility for all isolates was 12.6%, and higher proportions were observed for patients with healthcare exposure (community-acquired, community-onset 5.3%; healthcare-associated, community-onset 11.6%; hospital-onset 20.1%, p<0.01). Among all isolates, the nonsusceptible proportion was stable during the study (p = 0.12 by test for trend). However, there were increasing nonsusceptibility rates for community-acquired, community-onset isolates (4.7% in 2003–2007 and 5.6% in 2008–2013, p = 0.04 by test for trend), and there were no changes observed for other healthcare-associated, community-onset (p = 0.41) and hospital-onset (p = 0.28) isolates.

#### P. aeruginosa

**Figure 3:**
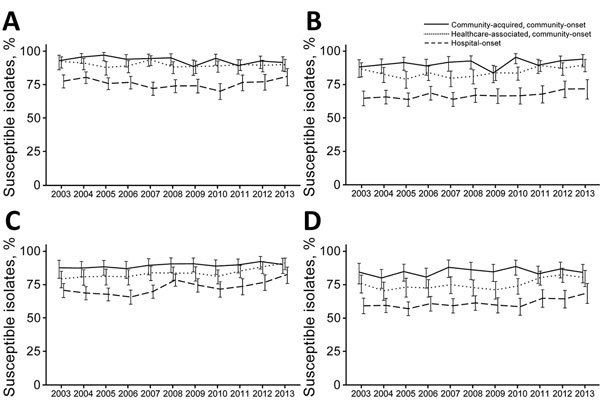
Trends of selected antimicrobial susceptibilities for *Pseudomonas aeruginosa* isolates from patients with bacteremia, Veterans Health Administration System, United States, 2003–2013. A) Antipseudomonal carbapenems, B) Antipseudomonal cephalosporins, C) Aminoglycosides, D) Antipseudomonal fluoroquinolones. Error bars indicate 95% CIs.

Antipseudomonal carbapenem nonsusceptibility was reported for 15.8% of all isolates (Figure 3). The rates for antipseudomonal carbapenem-nonsusceptible isolates were stable during the study (p = 0.66 by test for trend). For community-acquired, community-onset isolates, the proportion of nonsusceptible isolates increased (4.8% in 2003–2007 and 7.9% in 2008–2013, p = 0.03 by test for trend), but there was no significant trend for other categories (healthcare-associated, community-onset; p = 0.45 and hospital-onset; p = 0.40). Healthcare exposure was associated with higher rates of nonsusceptibility to antipseudomonal carbapenems (community-acquired, community-onset 6.6%; healthcare-associated, community-onset 10.0%; and hospital-onset 24.1%, p<0.01).

Healthcare exposure also increased nonsusceptibility to antipseudomonal extended-spectrum cephalosporins (community-acquired, community-onset 9.1%; healthcare-associated, community-onset 15.5%; hospital-onset 33.2%, p<0.01). Although 22.3% of all isolates were nonsusceptible, the proportion that were nonsusceptible decreased (24.8% in 2003–2007 and 20.0% in 2008–2013, p<0.01 by test for trend). Decreases were limited to healthcare-exposed patients (healthcare-associated, community-onset; p = 0.01 and hospital-onset; p = 0.04, but not community-acquired, community-onset; p = 0.16).

The overall rate for aminoglycoside nonsusceptible isolates was 20.6%, and nonsusceptible bacteria were more frequently isolated from patients with healthcare exposure (community-acquired, community-onset 10.7%; healthcare-associated, community-onset 16.4%; hospital-onset 28.0%, p<0.01). The overall trend was toward lower rates of nonsusceptible isolates (24.1% in 2003–2007 and 17.3% in 2008–2013, p<0.01 by test for trend). However, this trend of decreasing aminoglycoside nonsusceptibility rates was significant only for patients with healthcare exposure (community-acquired, community-onset, p = 0.14; healthcare-associated, community-onset, p<0.01; and hospital-onset, p<0.01).

The overall rate for antipseudomonal fluoroquinolone-nonsusceptible isolates was 29.6%; these isolates were obtained more frequently from patients who had healthcare-associated, community-onset (24.6%) and hospital-onset episodes (39.4%) than from patients who had community-acquired, community-onset episodes (15.4%, p<0.01). The proportion that was nonsusceptible decreased during the study (32.2% in 2003–2007 and 27.2% in 2008–2013, p<0.01 by test for trend), but this trend was significant only for patients with healthcare exposure (community-associated, p = 0.29; healthcare-associated, community-onset, p<0.01; and hospital- onset, p = 0.02).

#### *Acinetobacter* spp.

**Figure 4:**
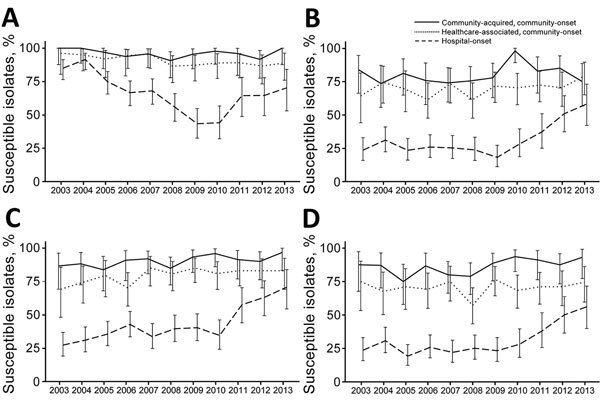
Trends of selected antimicrobial susceptibilities for *Acinetobacter* spp. isolates from patients with bacteremia, Veterans Health Administration System, United States, 2003–2013. A) Antipseudomonal carbapenems, B) Extended-spectrum cephalosporins, C) Aminoglycosides, D) Antipseudomonal fluoroquinolones. Error bars indicate 95% CIs.

Antipseudomonal carbapenem nonsusceptibility results were reported for 9.8% of the isolates (Figure 4). The proportion of nonsusceptible isolates was higher for isolates from patients with healthcare exposure (community-acquired, community-onset 4.1%; healthcare-associated, community-onset 9.4%; and hospital-onset 32.6%, p<0.01). The nonsusceptible proportion for hospital-onset isolates increased significantly from 15.0% in 2003 to 56.6% in 2009, but decreased to 29.7% in 2013 (trend in 2003–2009, p<0.01; trend in 2010–2013, p<0.01). The nonsusceptibility proportion for community-acquired, community-onset isolates showed a significant trend (p = 0.41), but this trend increased for healthcare-associated, community-onset isolates (5.4% in 2003–2007 and 12.1% in 2008–2013, p = 0.04).

Overall, 48.1% of isolates were nonsusceptible to extended-spectrum cephalosporins, and this proportion decreased during the study (54.3% in 2003–2007 and 42.5% in 2008–2013, p<0.01 by test for trend). This decrease was caused primarily by significant decreases in nonsusceptibility for hospital-onset isolates since 2009 (community-acquired, community-onset, p = 0.28; healthcare-associated, community-onset, p = 0.23; and hospital-onset, p<0.01). Extended-spectrum cephalosporin-nonsusceptible isolates were more frequent for patients with healthcare exposure (community-acquired, community-onset 19.6%; healthcare-associated, community-onset 30.1%; hospital-onset 70.4%, p<0.01).

Although the overall proportion of aminoglycoside-nonsusceptible isolates was 37.4%, the proportion considered nonsusceptible was higher as healthcare exposure increased (community-acquired, community-onset 9.6%; healthcare-associated, community-onset 19.9%; and hospital-onset 60.0%, p<0.01) The overall trend was toward decreasing rates of nonsusceptibility (45.7% in 2003–2007 and 29.9% in 2008–2013, p<0.01 by test for trend). The decrease was significant only for patients with healthcare exposure (community-acquired, community-onset, p = 0.08; healthcare-associated, community-onset, p = 0.04; and hospital-onset, p<0.01).

The overall proportion of isolates that were nonsusceptible to fluoroquinolones was 47.2%. Patients with healthcare exposure were more likely to be infected with nonsusceptible strains (community-acquired, community-onset 14.2%; healthcare-associated, community-onset 19.6%; hospital-onset 71.9%, p<0.01) There were significant decreases in nonsusceptibility to fluoroquinolones (54.3% in 2003–2007 and 41.0% in 2008–2013, p<0.01 by test for trend), which was observed for hospital-onset isolates only (community-acquired, community-onset, p = 0.07; healthcare-associated, community-onset HCA-CO, p = 0.73; hospital-onset, p< 0.01).

## Discussion

We analyzed 47,746 cases of gram-negative bacteremia in the VHA system over an 11-year period and report 3 major findings. First, for *E. coli*, rates of nonsusceptibility to aminoglycosides, fluoroquinolones, and extended-spectrum cephalosporins increased across all care settings. Second, for *Klebsiella* spp., rates of carbapenem nonsusceptibility increased across all care settings, and nonsusceptibility to other antimicrobial classes increased for community-onset isolates and was stable or decreased for isolates from patients with healthcare exposure. Third, for *P. aeruginosa* and *Acinetobacter* spp., rates of nonsusceptibility to aminoglycosides, fluoroquinolones, and extended-spectrum cephalosporins mostly decreased, but only for isolates from patients with healthcare exposure.

Overall incidence rates for gram-negative bacteremia were comparable with those from previous population-based studies from the United States and Europe (*3**,**13**,**20**–**22**,**30*). We recently reported major changes in hospital-onset bacteremia, which was possibly caused by expansion of horizontal infection control programs in the VHA system during this time (*15*). An additional finding in our more recent study was the decreased proportion of nonsusceptible bloodstream isolates across nearly all antimicrobial classes for *Klebsiella* spp., *P. aeruginosa*, and *Acinetobacter* spp. in patients with healthcare exposure. However, trends for community-onset bloodstream isolates were stable or showed increased nonsusceptibility.

Our findings suggest that efforts to improve infection control practices within the VHA might have had effects on antimicrobial susceptibilities for isolates from patients with healthcare exposure, although the extent of these effects needs to be determined. With increased nonsusceptibility rates among community-acquired, community-onset isolates, we generally expect upward trends of nonsusceptibility rates for healthcare-associated and hospital-onset isolates caused by increased colonization pressure (*31*,*32*). However, our results showed improvements in nonsusceptibility rates for isolates of *Klebsiella* spp., *P. aeruginosa*, and *Acinetobacter* spp. in hospital-onset and healthcare-associated, community-onset infections, which might suggest successful interruption of transmission in inpatient settings. Infection control programs within the VHA have been expanded over the past 10 years, especially since introduction of the Methicillin-Resistant *Staphylococcus aureus* Prevention Initiative in 2007 (*33*). Our recent study showed a substantial decrease in nosocomial, gram-negative bacteremia after implementation of this initiative, which suggests a collateral benefit beyond the originally intended scope of this initiative (*15*). In addition, the VHA released a mandatory policy for hand hygiene practices in 2011 (*34*).

Antimicrobial stewardship efforts within the VHA, which have been evolving over the past 10 years, might have contributed to increased antimicrobial susceptibilities. The National VA Antimicrobial Stewardship Task Force, established in 2011, has been leading systemwide educational efforts, and a mandate for inpatient stewardship was enacted in 2014. However, even before these broad interventions, facilities were actively performing stewardship. According to a survey of VHA facilities conducted by the VA Healthcare Analysis and Information Group, 86 (66.2%) of 130 VHA facilities had either a formal or informal antimicrobial stewardship policy in place in 2012 (*35*). At the time of the survey, 14 (16.3%) of these facilities had a stewardship policy in place for 2 years, 7 (8.1%) for 3 years, and 33 (38.4%) for >4 years.

We speculate that increased rates of antimicrobial susceptibility were not observed for *E. coli* healthcare-associated, community-onset and hospital-onset isolates because patients were probably colonized with *E. coli* before admission and these commensal strains might have developed or acquired resistance in the community setting. Antimicrobial drug resistance can develop in *E. coli* by its exposure to retail meat (*36*) and antimicrobial drug exposure outside VHA hospitals. Antimicrobial stewardship efforts within the VHA, like most healthcare systems, have typically not focused on outpatients. In addition, many non-VHA hospitals, which might have cared for patients in this cohort but were not captured by our analysis, did not have stewardship programs during this study (*37*,*38*).

A notable exception to these trends is increased rates of carbapenem nonsusceptibility for *Klebsiella* spp. in community and hospital settings. The increase in carbapenem-resistant *Klebsiella* spp. was also reported outside the VHA (*39*–*41*). This observation deserves attention because it might indicate a lapse of infection control efforts at the VHA. The VHA implemented internal guidelines for carbapenem-resistant *Enterobacteriaceae* to address this issue in 2015, and additional research is needed to determine whether this intervention has had any positive effect on this major threat (*42*).

The strength of our study was inclusion of all bacteremia episodes from the entire VHA system, which enabled us to perform population-based analysis, including diverse care settings, for wide geographic regions. Our study demonstrated the advantage of an integrated health informatics infrastructure with a clinical data warehouse within the VHA system. To accurately monitor the burden and trends of antimicrobial resistance, more investments in data integration informatics infrastructure need to be made.

This study had several limitations. First, most patients seen within the VHA system were male. Therefore, results might not be generalizable to the rest of the population. Second, because information was collected through patient medical records, detailed microbiological information, such as break points of antimicrobial susceptibility testing, molecular typing, or mechanisms of resistance, was often not available. Third, the Clinical and Laboratory Standards Institute (CLSI) revised its break point recommendations for extended-spectrum cephalosporins and carbapenems in 2010 (*43*), and antimicrobial susceptibility trends in the later part of the study might have been affected by this change. However, the FDA did not revise its recommendations to match those of the CLSI until 2013 (*44*). Because most microbiology laboratories in the VHA system use commercial systems that are regulated by the FDA for susceptibility testing, it is less likely that the change of CLSI break points substantially affected our observations. Fourth, this study focused on nationwide overall trends, and results might reflect local trends in each specific region. Previous studies showed geographic variabilities in incidence rates and antimicrobial susceptibilities for gram-negative bacteria (*45*–*47*). We also recently reported regional variations in fluoroquinolone nonsusceptibilities for *E. coli* isolates and plan to analyze geographic variabilities for other organisms and antimicrobial agents in future studies (*48*). Fifth, resistance can develop in gram-negative bacteria during patient therapy, and assessing susceptibilities for the primary isolate from each episode might underestimate the prevalence of nonsusceptibilities. Because there were diversities in reporting practices of susceptibilities for subsequent isolates across the system, it was not feasible to collect information for those isolates in a standardized manner.

In conclusion, we observed an increase in antimicrobial drug nonsusceptibility for *E. coli* in all healthcare exposure categories and stable or decreased rates of nonsusceptibility for *Klebsiella* spp., *P. aeruginosa*, and *Acinetobacter* spp. in patients with healthcare exposure. The extent to which these findings might reflect VHA infection control and antimicrobial stewardship activities remains to be determined. Despite encouraging decreases in nonsusceptibility for pathogenic gram-negative bacteria in acute-care hospital settings, increased attention to infection prevention and antimicrobial stewardship is needed for outpatient and nonacute inpatient settings.

Technical Appendix Additional information on antimicrobial nonsusceptibility of gram-negative bloodstream isolates, Veterans Health Administration System, United States, 2003–2013.
