# Supplementary material for: Antimicrobial Nonsusceptibility of Gram-Negative Bloodstream Isolates, Veterans Health Administration System, United States, 2003–2013
Source: Emerg Infect Dis. 2017 Nov;23(11):1815–25. doi: 10.3201/eid2311.161214 (PMC5652419; doi:10.3201/eid2311.161214)
Supplement: Technical Appendix — Additional information on antimicrobial nonsusceptibility of gram-negative bloodstream isolates, Veterans Health Administration System, United States, 2003–2013. [file 16-1214-Techapp-s1.pdf]

Article DOI: https://doi.org/10.3201/eid2311.161214

Antimicrobial Nonsusceptibility of Gram-Negative Bloodstream Isolates, Veterans Health Administration System, United States, 2003–2013

Technical Appendix

Technical Appendix Table 1. Antimicrobial classes and drugs used for determining antimicrobial nonsusceptibility of *Escherichia coli* and *Klebsiella* spp. bloodstream isolates, Veterans Health Administration System, United States, 2003–2013

Class	Drug
Aminoglycosides	Gentamicin
	Tobramycin
	Amikacin
Carbapenems	Ertapenem
	Imipenem
	Meropenem
	Doripenem
Extended-spectrum cephalosporins	Ceftriaxone
	Cefotaxime
	Ceftazidime
	Cefepime
Fluoroquinolones	Ofloxacin
	Ciprofloxacin
	Levofloxacin
	Gatifloxacin
	Moxifloxacin

Technical Appendix Table 2. Antimicrobial classes and drugs used for determining antimicrobial nonsusceptibility of *Pseudomonas aeruginosa* bloodstream isolates, Veterans Health Administration System, United States, 2003–2013

Class	Drug
Aminoglycosides	Gentamicin
	Tobramycin
	Amikacin
Antipseudomonal carbapenems	Imipenem
	Meropenem
	Doripenem
Antipseudomonal cephalosporins	Ceftazidime
	Cefepime
Antipseudomonal fluoroquinolones	Ofloxacin
	Ciprofloxacin
	Levofloxacin
	Gatifloxacin

Technical Appendix Table 3. Antimicrobial classes and drugs used for determining antimicrobial nonsusceptibility of *Acinetobacter* spp. bloodstream isolates, Veterans Health Administration System, United States, 2003–2013

Class	Drug
Aminoglycosides	Gentamicin
	Tobramycin
	Amikacin
Antipseudomonal carbapenems	Imipenem
	Meropenem
	Doripenem
Antipseudomomal fluoroquninolones	Ofloxacin
	Ciprofloxacin
	Levofloxacin
	Gatifloxacin
Extended-spectrum cephalosporins	Ceftriaxone
	Cefotaxime
	Ceftazidime
	Cefepime

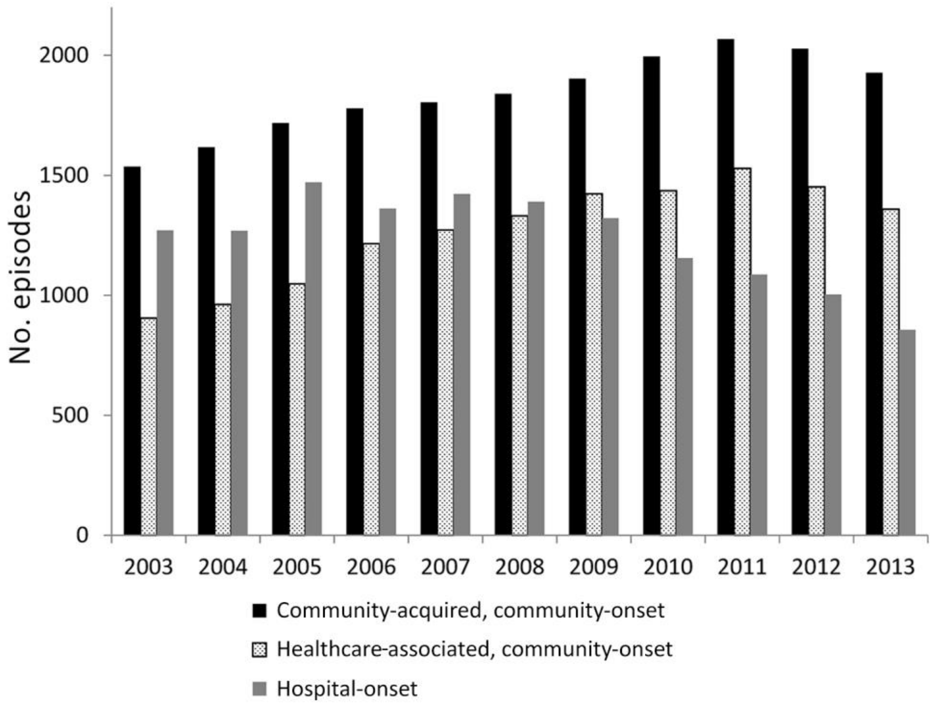

Technical Appendix Figure 1. No. episodes of gram-negative bacteremia, by place of acquisition, Veterans Health Administration System, United States, 2003–2013.

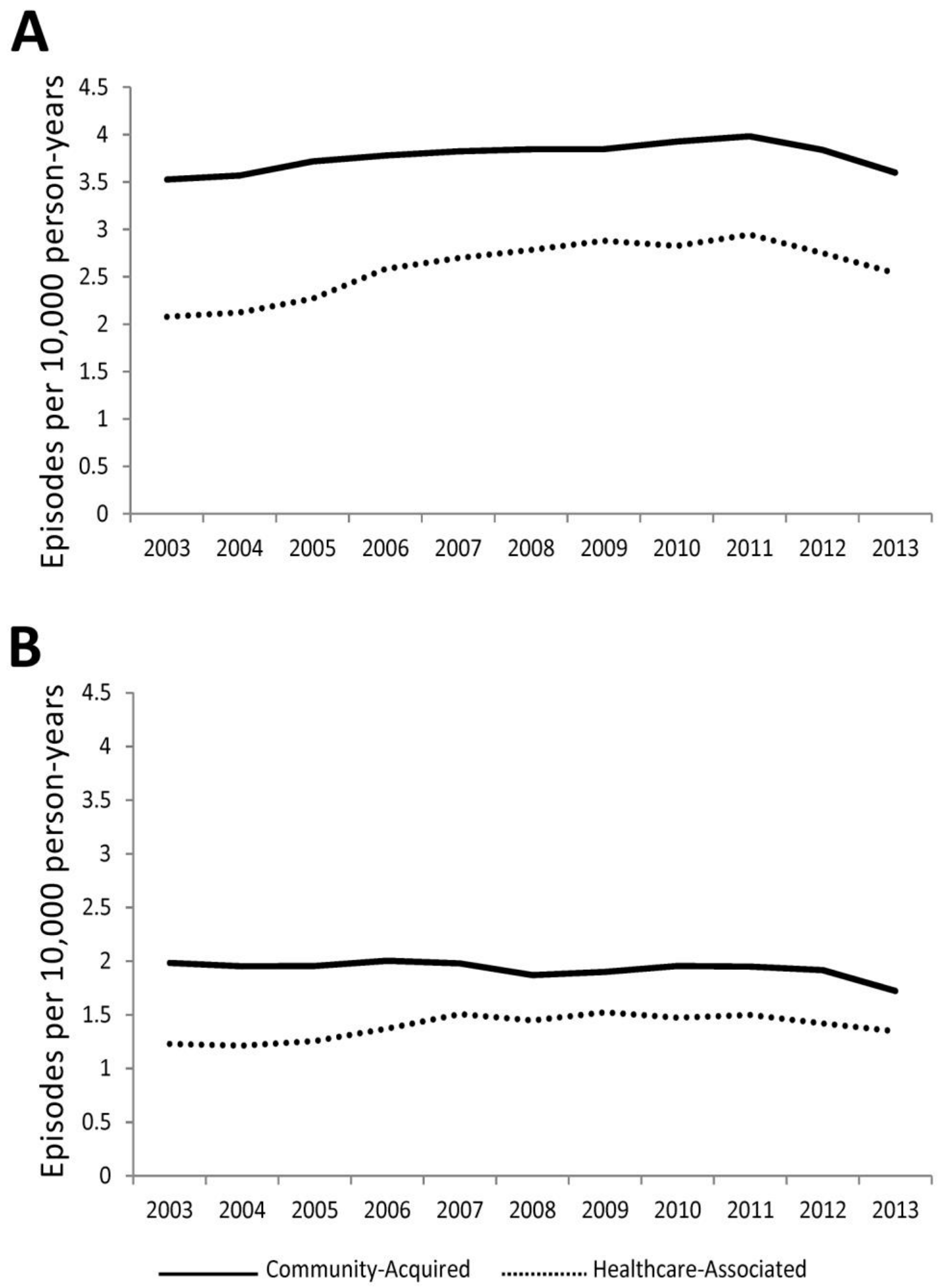

Technical Appendix Figure 2. A) Crude and B) age-standardized incidence rates for community-onset gram-negative bacteremia, Veterans Health Administration System, United States, 2003–2013.

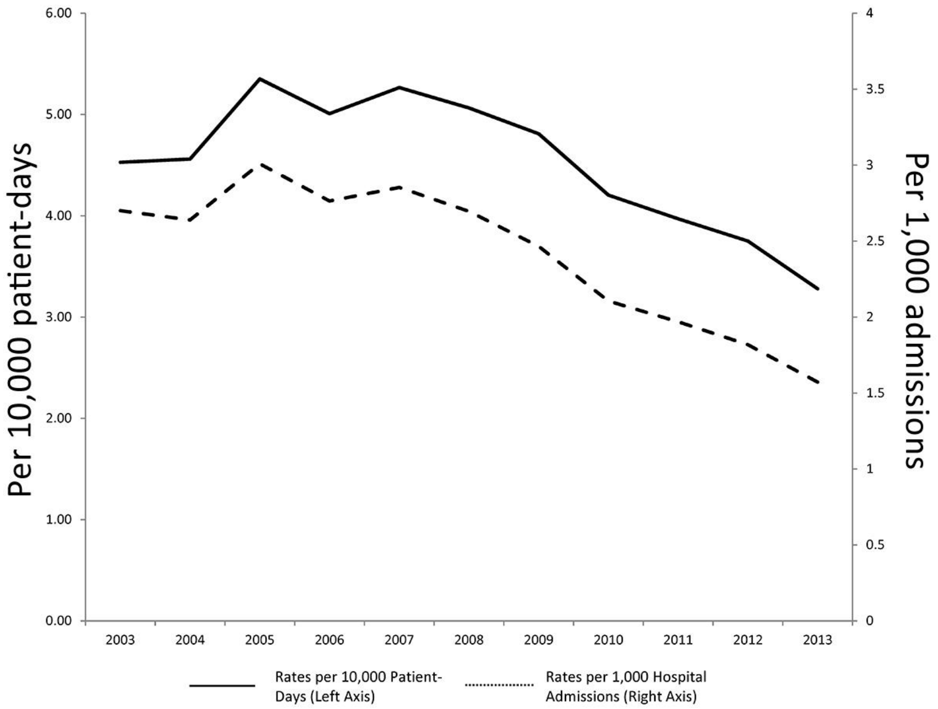

Technical Appendix Figure 3. Incidence rates for hospital-onset gram-negative bacteremia, Veterans Health Administration System, United States, 2003–2013.

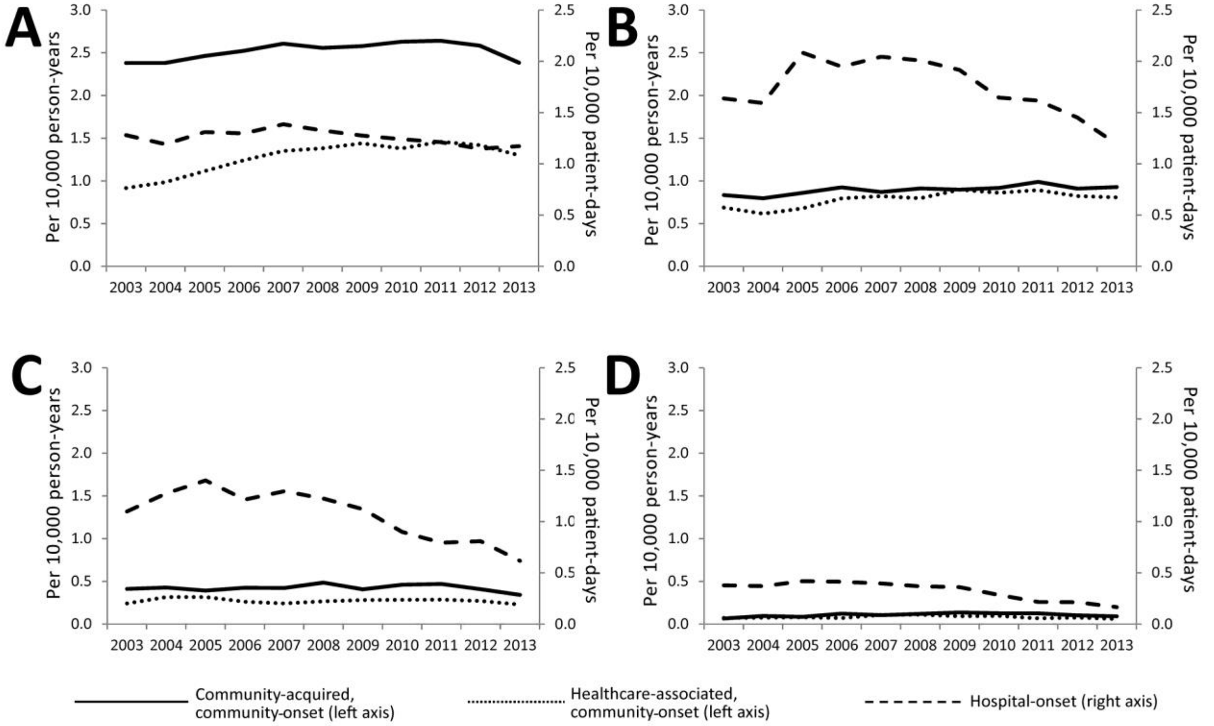

Technical Appendix Figure 4. Incidence rates for A) *Escherichia coli*, B) *Klebsiella* spp., C) *Pseudomonas aeruginosa*, and D) *Acinetobacter* spp. bacteremia, Veterans Health Administration System, United States, 2003–2013.
